# Evaluation of a training program for health care workers to improve the quality of care for rape survivors: a quasi-experimental design study in Morogoro, Tanzania

**DOI:** 10.3402/gha.v9.31735

**Published:** 2016-07-14

**Authors:** Muzdalifat Abeid, Projestine Muganyizi, Rose Mpembeni, Elisabeth Darj, Pia Axemo

**Affiliations:** 1 Department of Women's and Children's Health, International Maternal and Child Health (IMCH), Uppsala University, Uppsala, Sweden; 2 Department of Obstetrics/Gynecology, Muhimbili University of Health and Allied Sciences (MUHAS), Dar es Salaam, Tanzania; 3 Department of Epidemiology and Biostatistics, Muhimbili University of Health and Allied Sciences (MUHAS), Dar es Salaam, Tanzania; 4 Department of Public Health and General Practice, Norwegian University of Science and Technology, Trondheim, Norway

**Keywords:** sexual violence, health care workers, quasi-experimental design, training, Tanzania

## Abstract

**Background:**

Sexual violence against women and children in Tanzania and globally is a human rights violation and a developmental challenge.

**Objective:**

The aim of this study was to assess the impact of training health professionals on rape management. The specific objectives were to evaluate the changes of knowledge and attitudes toward sexual violence among a selected population of health professionals at primary health care level.

**Design:**

A quasi-experimental design using cross-sectional surveys was conducted to evaluate health care workers’ knowledge, attitude, and clinical practice toward sexual violence before and after the training program. The study involved the Kilombero (intervention) and Ulanga (comparison) districts in Morogoro region. A total of 151 health professionals at baseline (2012) and 169 in the final assessment (2014) participated in the survey. Data were collected using the same structured questionnaire. The amount of change in key indicators from baseline to final assessment in the two areas was compared using composite scores in the pre- and post-interventions, and the net intervention effect was calculated by the difference in difference method.

**Results:**

Overall, there was improved knowledge in the intervention district from 55% at baseline to 86% and a decreased knowledge from 58.5 to 36.2% in the comparison area with a net effect of 53.7% and a *p*-value less than 0.0001. The proportion of participants who exhibited an accepting attitude toward violence declined from 15.3 to 11.2% in the intervention area but increased from 13.2 to 20.0% in the comparison area.

**Conclusions:**

Training on the management of sexual violence is feasible and the results indicate improvement in healthcare workers’ knowledge and practice but not attitudes. Lessons learned from this study for successful replication of such an intervention in similar settings require commitment from those at strategic level within the health service to ensure that adequate resources are made available.

## Introduction

Sexual violence against women and children globally is a human rights violation and a developmental challenge. Worldwide, experience of sexual violence is estimated to be 14–25% (1–4). In Tanzania, sexual violence against women and children is a serious public health concern. Two in 10 Tanzanian women would have experienced sexual violence at some point (5). This figure includes women for whom sexual initiation was forced against their will, of whom 1 in 10 women report (5). Children under the age of 18, 28% of girls and 13% of boys, are reported to have experienced sexual violence (6).

Women who have experienced sexual violence have higher incidences of gynecological disorders such as sexually transmitted infections (STIs), including HIV, unwanted pregnancies, and chronic pelvic pain (7–9). Sexual violence has been specifically linked to an increased risk of HIV and AIDS for the exposed women (10). Likewise, women experiencing sexual violence more often present with posttraumatic stress disorders, depression, anxiety, and suicidal ideation (8–11).

Health care professionals have a pivotal role to play in supporting sexual violence survivors as they meet these clients in their daily work (12). For instance, in order to reduce the complications of sexual violence, standard care protocols that include medical investigations; treatment of injuries; prevention of STIs, including HIV; prevention of pregnancy using emergency contraception (EC); and psychological counseling are recommended at the health facility level (13).

Traditionally, however, the quality of care for women experiencing sexual violence has been poor worldwide (14). A study of midwives revealed that a majority is often reluctant to make adequate inquiries about gender-based violence (GBV); therefore, sexual violence remains a hidden problem in most health care settings (15). Surveys to determine levels of training, knowledge, and attitudes among health care workers (HCWs) have shown that most health care professionals have positive attitudes and are sympathetic toward clients, acknowledging that GBV, including sexual violence, is a health care issue; however, they often lack fundamental knowledge about issues surrounding GBV and about appropriate agencies that can offer help (16–19).

Training has an effect on practitioners’ knowledge, attitude, and clinical practice. It gives the health professionals an opportunity to update their knowledge about gender issues and improve the care they provide to sexual violence survivors (20, 21). There is limited information in low- and middle-income countries about the effectiveness of the HCW training programs. Of late, there has been greater focus on the formal evaluation of interventions in health care settings designed to ameliorate the harmful effects of violence against women. The few studies in Africa that have looked at the impact of training programs have reported a positive impact on the support given by health workers (20, 21).

Tanzania, through the Ministry of Health and Social Welfare (MOHSW), has developed a clinical training curriculum for HCWs which includes GBV screening protocols. Although there is a growing awareness of GBV in the Tanzanian society and increased efforts at policy level to address the issue, survivors’ access to health care remains limited, and the training of service providers is yet to be rolled out adequately. The aim of this study was to assess the impact of the training of health professionals. Our hypothesis was that training would increase the HCWs’ knowledge and skills, which would eventually improve their provision of care of sexual violence survivors. The specific objectives were to evaluate the changes in knowledge and attitudes toward sexual violence against women and children, including their management, in a selected population of health professionals at primary health care level.

## Methods

### Study design

This was an intervention study which employed pre- and post-comparison cross-sectional surveys to evaluate HCWs’ knowledge, attitude, and clinical practice related to sexual violence before and after a training program. The research involved two arms: the Kilombero district (intervention) and the Ulanga district (comparison).

### Study setting

The public health system, administered by the MOHSW of Tanzania, is decentralized. Health dispensaries provide the most basic level of care and can assist a survivor with first aid for minor injuries. Most often they will refer sexual violence cases to a higher-level health facility with more comprehensive services. Health centers offer the next level of care and can treat a wider range of injuries. While district hospitals offer both inpatient and outpatient care and some basic laboratory testing. Kilombero and Ulanga districts are both located in the Morogoro region, southwest of Dar es Salaam. Both districts are comparable in sociodemographic characteristics as well as in economic-earning activities that include farming and fishing. The total population of the Kilombero district is 416,401, and it has a total of five divisions, each of which has one health center. It has one designated district referral hospital (22). The Ulanga district has a total population of 234,219, with seven divisions, including three health centers and two hospitals (23). The prevalence of sexual violence for both districts is not known. Based on health centers and hospital records, the number of rape cases reported is 2–5 per month in a health facility. All health centers and the district hospitals in both areas provide voluntary counseling and testing (VCT) for HIV, and they also offer care and treatment for HIV/AIDS. An inventory checklist prior to the fieldwork was developed and used to collect information about the services offered at the selected facilities. The checklist contained questions about the number of staff, number of rape cases per month, services on-site, referral for rape survivors, the guidelines and protocols used, and the training offered. The information gathered indicated that, at baseline, for intervention and comparison areas, most post-rape care for survivors was delivered by a doctor, with the nursing role confined primarily to assisting the doctor. The post-rape care was basically composed of offering VCT and post-exposure prophylaxis (PEP) for free. For other laboratory investigations, medications for STI, and EC, if available, the patient was obligated to pay. There were no written guidelines or protocols for the management of rape and child sexual abuse. This led to inadequate documentation of the history and examination, and improper forensic evidence collection. The majority of health staff approached had not received any formal training on GBV but had some exposure through the continuous medical education at the hospital. Selected HCWs from the district hospital and the five health centers in the intervention area were recruited for the training program.

### Study participants

The study participants included HCWs from the selected facilities in the two districts that were available at the time of this study. In this study, the term, ‘HCW’ refers to all levels of health care education (certificate, diploma, and degree) to include clinicians, namely medical officer (MD), assistant medical officers (AMOs), clinical officers (COs), nurses and medical attendants working at the health centers and hospitals in the study areas. In Tanzania, the majority of clinicians working in the rural settings include COs and AMOs. The clinical officer training program lasts 3 years and experienced COs may enroll for an advanced 2-year diploma in clinical medicine (which also includes 3 months in surgery and 3 months in obstetrics) to qualify as medical licentiates (AMOs). The total number of HCWs in each health center in the intervention area at the time of the study was, on average, 15. The intention was to include as many HCWs as possible. A total of 50 HCWs from the five health centers that were available participated in the survey and training. The number of HCWs at the district hospital was 320. However, from the district hospital, 50 of a total of 89 HCWs from the outpatient department and the departments of pediatrics and gynecology, and the laboratory were randomly selected for participation in the survey and training. The HCWs in these departments were selected as they are more likely to meet survivors of sexual violence. The selection of participants from the comparison area was purposive with priority given to doctors and nurses who were available and willing to participate only in the survey. Although the number of doctors working in the district was few, more certificate-level medical attendants were included in the control than in the intervention district.

### Sample and data collection

A sample of 151 HCWs (intervention 98 vs. 53 comparison) at baseline and 169 (intervention 89 vs. 80 comparison) at final assessment participated in the surveys. In the intervention district, 100 HCWs were trained but at baseline only 98 HCWs participated in the survey; two HCWs did not return the questionnaires. In the final assessment, however, 89 HCWS participated in the survey as nine HCWS had either left for further studies or were transferred to another facility in another region. It was a self-administered survey. The questionnaire was adapted from a study conducted in Vietnam on improving health care response to GBV (24). It comprises three sections: respondent's profile; actual knowledge, attitude, and practice toward sexual violence; and recommendations for future improvement in responding to sexual violence survivors. The same questionnaire was used at baseline in December 2012–January 2013 and after the training program in February–March 2014. Baseline information was collected just before the training session commenced. Members of the research team introduced the objective of the survey as well as the confidentiality of the answers and invited attending practitioners to anonymously self-complete the questionnaire after obtaining verbal consent. The study coordinator, who is also the first author (MA), checked all questionnaires to ensure their accuracy and completeness. The same HCWs from the intervention area who completed the baseline questionnaire were recruited for the training program and later were followed up at final assessment survey in 2014. For the comparison area, where no training took place, not all of the HCWs who completed the baseline survey participated in the final assessment.

### Training program

The training took place between February and April 2013. The objective of this training was to improve knowledge of sexual violence and its clinical management and provide prompt medical support to rape survivors. A total of 100 HCWs from the five health centers and district hospital in the intervention district participated in the training in three batches. A private hall was hired to conduct the training program. This was a 5-day training program which was conducted using the WHO/UNHCR guidelines and the National GBV management guidelines (13, 25). The first author, MA; the co-author, PM; and the local gynecologist from the district hospital facilitated the training. Participatory learning methods, including lectures, discussions, group work, and case studies/scenarios were used. A warm-up session on ‘Building Awareness on Gender’ preceded the actual training which covered the basic concepts of gender and the commonly accepted norms. The topics covered for the training included:

**Table:** 

**Topics**
**Introduction to GBV:** Its magnitude, types of GBV, contributing factors to GBV, and national laws and policy on GBV
**Responsibilities of HCWs:** Provision of medical care, collection of forensic evidence, refer to other care providers and services
**Obtaining consent from the survivor:** Importance of giving information before performing an exam, key steps in obtaining consent
**Introduction to survivor-centered medical history:** Basic interview techniques, important components of a medical history
**Introduction to examination and collection of forensic evidence:** Use of survivors’ history to guide the exam, collection of forensic evidence
**Treatment for consequences of rape:** General wound care, treatment of STIs, including HIV, provision of EC as per national guideline
**Psychological support for survivors:** Emphasis on basic counseling skills, refer for professional support
**Medical care of the child survivor:** Special attention to a child, care of a child, creating a safe environment for a child

Estimated staff completion of the training is 90% for doctors and 50% for nurses. After the training of HCWs, all of the five health centers and the district hospital in the intervention area were provided with the National Management Guidelines and pre-packed rape kits which included supplies for forensic evidence collection, medications for prevention of STI, pregnancy tests, and EC and HIV testing reagents. On-site services included medical care, documentation of injuries, referral to a higher-level hospital, or external referral for police investigations and legal support. Rape registry books were provided to the health facilities in both the intervention and comparison areas for documentation of all rape cases the HCWs attended before the intervention and throughout the whole study period. In the facilities located in the comparison area, services were provided as per normal routine. The post-training survey was conducted in February–March 2014 for both the districts using the same questionnaire.

### Analysis

Data were double-entered into EpiData 3.0 and analyzed using Statistical Package for Social Sciences (SPSS) version 21 for the calculation of frequencies as percentages, and averages as means and standard deviations (SDs). The Chi-square test was used for nominal and categorical data and, if tables had at least 25% of cells with fewer counts than expected, significance was estimated using Fisher's exact test. Each question was categorized as yes/no. The aim of the statistical analyses was to estimate the effects of intervention on the proportion of yes answers. The intervention effect was estimated as the difference between intervention and comparison groups regarding changes in proportions from baseline to end line. This effect is a linear combination of four independent estimates. *P* values from a *Z-*test and 95% confidence intervals for the intervention effect were calculated based on a normal distribution assumption. *P*<0.05 was considered a statistically significant result (26). Statistical analyses were performed with SAS version 9.4 (SAS Institute Inc., Cary, NC, USA).

### Outcome variables

The main outcome measures were knowledge, attitude, and clinical practice of HCWs. A binary outcome variable was created for knowledge and attitude. The total sum score for knowledge questions was 16 points. The scale was dichotomized using the two-third rule to categorize respondents with a score of 0–66% as having incorrect knowledge on sexual violence, and all those who score 67–100% as having correct knowledge on sexual violence. The total sum score for attitude questions was 14 points. Applying the same two-third rule, the scale was dichotomized to obtain respondents with accepting (equal or above 67%) and non-accepting attitudes (below 67%) toward sexual violence. The program effects were compared using the composite score in the pre-intervention and post-intervention measures for each district.

### Ethical clearance

This study is part of a larger study on sexual violence against women and children in the Morogoro region, Tanzania. The institutional review board (IRB) of the Muhimbili University of Health and Allied Sciences (MUHAS) granted ethical approval. Written permission for conducting the study was obtained from Kilombero and Ulanga municipalities. Ethical guidelines for researching violence against women approved by WHO/CIOM (27) were followed. This included asking for informed written consent of HCWs after reading the consent form approved by the IRB. However, some HCWs preferred to keep the forms and provided verbal consent to avoid being identified. The verbal consent was denoted on the questionnaire. We ensured confidentiality in the meeting between the survivors and HCWs. Training of the HCWs included sessions to assure proper counseling and referral of women and children exposed to violence. Caring for sexual violence may be emotionally sensitive for HCWs. The research team arranged for a professional counselor to support them if needed.

## Results

### Sample characteristics

The sample included 151 HCWs at baseline and 169 at final assessment. The mean age was 37.7 (10.8) and 37.9 (8.3) years in the intervention and comparison groups, respectively, at baseline, ranging from 18 to 60 years. With regard to occupation, the majority of the survey participants were nurses at baseline in the intervention and comparison areas (48% vs. 52.8%), with the rest being clinicians and other support staff. At both baseline and final assessment, HCWs who participated in the intervention and comparison areas were similar in terms of age, gender, marital status, and work experience. However, the intervention area had a significantly higher proportion of physicians than the comparison area at baseline as well as at final assessment, as shown in Table 1.

**Table 1:** Demographic characteristics of HCWs involved in the intervention and comparison areas, at baseline and final assessment

	Baseline (2012)			Final (2014)		
		
Variable	Intervention *n*=98 (%)	Comparison *n*=53 (%)	*p*	Intervention *n*=89 (%)	Comparison *n*=80 (%)	*p*
Cadre						
MD	15 (15.3)	4 (7.6)	0.077	9 (10.1)	4 (5.0)	<0.001
AMO/CO	23 (23.5)	19 (35.8)		22 (24.7)	7 (8.8)	
Nurses	47 (47.9)	28 (52.8)		45 (50.6)	31 (38.7)	
Other	13 (13.3)	2 (3.8)		13 (14.6)	38 (47.5)	
Sex						
Male	39 (39.8)	22 (41.5)	0.838	31 (34.8)	27 (33.8)	0.882
Female	59 (60.2)	31 (58.5)		58 (65.2)	53 (66.2)	
Age in Years						
Mean [SD]	37.7 [10.8]	37.9 [8.3]	0.909	40.0 [11.2]	37.7 [12.9]	0.211
Marital status						
Married and ever married	56 (57.1)	36 (67.9)	0.195	59 (66.3)	54 (67.5)	0.868
Single	42 (42.9)	17 (32.1)		30 (33.7)	26 (32.5)	
Work experience in years						
Mean [SD]	11.2 [10.6]	11.4 [9.2]	0.879	14 [11.5]	12.6 [11.9]	0.392

HCWs, health care workers; MD, medical doctors; AMO/CO, assistant medical officer/clinical officer.

### Knowledge

Knowledge of HCWs on sexual violence at baseline in the 2012 survey is compared with findings from the 2014 survey in Table 2. Overall, there was improved knowledge in the intervention district from 55% at baseline to 86%, and a decreased knowledge from 58.5 to 36.2% in the comparison area with a net effect of 53.7% (95% CI: 32.2–75.1, *p*<0.0001), as shown in Table 2.

**Table 2:** Health care workers with correct knowledge on sexual violence in the intervention and comparison areas at baseline and final assessment

	Intervention			Comparison						
						
	Pre *n*=98 (%)	Post *n*=89 (%)	Estimates of change (%)	Pre *n*=53 (%)	*n*=80 (%)	Estimates of change (%)	NIE	CI95 Lower	CI95 Upper	*p*
Care of rape survivors										
HCWs suspicious of rape are obliged to interrogate the issue	54 (55.1)	34 (38.2)	−16.9	44 (83.1)	23 (28.7)	−54.3	37.4	16.8	57.9	0.0003
Rape of women and children should be treated as emergency	76 (77.6)	81 (91.0)	13.5	48 (90.5)	53 (66.3)	−24.3	37.8	20.8	54.7	**<0.0001**
HCWs have a legal obligation to give forensic evidence in court	54 (55.2)	79 (88.8)	33.7	35 (66.0)	52 (65.0)	−1	34.7	13.8	55.6	0.0009
Causes leading to sexual violence										
Influence of sex movies	80 (81.6)	74 (83.1)	1.5	42 (79.2)	60 (75.0)	−4.2	5.8	−12.9	24.4	0.5364
Influence of alcohol, drugs	94 (95.9)	85 (95.5)	−0.4	50 (94.3)	75 (93.8)	−0.6	0.2	−10.1	10.5	0.9726
Change in traditional values	72 (73.5)	73 (82.0)	8.6	37 (69.8)	66 (82.5)	12.7	−4.1	−23.7	15.4	0.6724
Health consequences										
Impact on women's health	80 (81.6)	79 (88.8)	7.1	53 (100)	68 (85.0)	−15	22.1	9	35.2	0.0007
Impact on women's mental health and psychology	91 (92.9)	79 (88.8)	−4.1	49 (92.5)	62 (77.5)	−15	10.9	−3.8	25.5	0.1382
Impact on women's reproductive health	85 (86.7)	77 (86.5)	−0.2	49 (92.5)	59 (73.8)	−18.7	18.5	2.6	34.4	0.0199
Impact on the long-term development	61 (62.2)	71 (79.8)	17.5	43 (81.1)	52 (65.0)	−16.1	33.7	13.6	53.7	0.0008
Perpetrators of sexual violence										
Acquaintances	38 (38.8)	83 (93.3)	54.5	27 (50.9)	28 (35.0)	−15.9	70.4	49.6	91.3	**<0.0001**
Close relatives	30 (30.6)	85 (95.5)	64.9	20 (37.7)	40 (50.0)	12.3	52.6	32.3	73	**<0.0001**
Awareness of National GBV management guideline	49 (50.0)	86 (96.6)	46.6	21 (39.6)	29 (36.2)	−3.4	50	29.5	70.5	**<0.0001**
Agencies/organizations that provide support to the victims of violence										
Police	70 (71.4)	80 (89.9)	18.5	29 (54.7)	34 (42.5)	−12.2	30.7	9.7	51.6	0.0034
Judicial offices	49 (50.0)	67 (75.3)	25.3	20 (37.7)	27 (33.8)	−4	29.3	7.3	51.2	0.0076
Government authorities	53 (54.1)	68 (76.4)	22.3	12 (22.6)	19 (23.8)	1.1	21.2	0.9	41.5	0.0363
Composite scores										
Correct knowledge	54 (55.1)	77 (86.5)	31.4	31 (58.5)	29 (36.2)	−22.3	53.7	32.2	75.1	**<0.0001**

CI, confidence interval; GBV, gender-based violence; HCWs, health care workers; NIE, net intervention effect (difference in intervention area from baseline to endline minus difference in comparison area from baseline to endline).

### Attitude

On comparing the 2012 and the 2014 HCW surveys, Table 3 shows that the proportion of participants who exhibited an accepting attitude toward violence declined from 15.3 to 11.2% in the intervention area but increased from 13.2 to 20.0% in the comparison area. However, the observed overall changes in the intervention and comparison areas were not statistically significant with a net effect of −10.9% (95% CI: −27.2–5.5, *p*=0.1845). Marked decline in accepting attitudes among HCWs was noted in the items that were related to justifying men beating their partners for whatever reason.

**Table 3:** Health care workers’ acceptance of violence against women norms in the intervention and comparison areas at baseline and final assessment

	Intervention			Comparison						
						
	Pre *n*=98 (%)	Post *n*=89 (%)	Estimates of change (%)	Pre *n*=53 (%)	Post *n*=80 (%)	Estimates of change (%)	NIE	CI95 Lower	CI95 Upper	*p*
Justification of a man beating his partner if she:										
Does not fulfill the domestic duties as expected	13 (13.3)	2 (2.2)	11	8 (15.1)	13 (16.2)	−1.1	12.2	−2.8	27.2	0.1045
Is addicted to alcohol and drugs	15 (15.3)	7 (7.9)	7.4	13 (24.5)	19 (23.8)	0.7	6.7	−11.2	24.6	0.4567
Contradicts her husband's opinions	14 (14.3)	5 (5.6)	8.7	10 (18.9)	15 (18.8)	0.1	8.6	−7.9	25	0.2976
Insults and abuses her spouse	13 (13.3)	5 (5.6)	7.6	16 (30.2)	27 (33.8)	−3.6	11.2	−7.4	29.8	0.2287
Does not satisfy her husband's sexual demand	21 (21.4)	6 (6.7)	14.7	5 (9.4)	17 (21.2)	−11.8	26.5	10.7	42.3	0.0008
Has extra-marital relations	18 (18.6)	7 (7.9)	10.5	19 (35.8)	22 (27.5)	8.3	2.2	−17.2	21.5	0.8235
Sexual violence										
Forcing women to have more babies/pregnancies	20 (20.4)	14 (15.7)	−4.7	12 (22.6)	32 (40.0)	17.4				0.0244
Preventing women from using contraceptives	35 (35.7)	20 (22.5)	−13.2	17 (32.1)	32 (40.0)	7.9	−21.2	−42.7	0.4	0.0491
Forcing to have a sexual intercourse when she doesn't want to	26 (26.5)	17 (19.1)	−7.4	8 (15.1)	26 (32.5)	17.4	−24.8	−43.8	−5.9	0.0089
Rape and sexual assault must be considered violence against women	10 (10.2)	8 (9.0)	1.2	2 (3.8)	7 (8.8)	−5	6.2	−5.8	18.2	0.3010
Rape and sexual assault against children or any type of sexual intercourse with children must be considered acts of violence	11 (11.2)	7 (7.9)	3.4	0 (0.0)	10 (12.5)	−12.5	15.9	4.5	27.2	0.0053
A woman's prior sexual relationship has a lot to do with rape	25 (25.5)	44 (49.4)	−23.9	19 (35.8)	38 (47.4)	−11.6	−12.3	−10	34.5	0.2698
Rape does not hurt women who are sexually experienced	10 (10.2)	14 (15.7)	−5.5	4 (7.6)	15 (18.7)	−11.1	5.6	−20.8	9.5	0.4530
Rape always leaves obvious signs of injuries	49 (50.0)	36 (40.5)	9.5	33 (62.2)	45 (56.3)	5.9	3.6	−26.3	19.2	0.7560
Composite scores										
Accepting attitude	15 (15.3)	10 (11.2)	−4.1	7 (13.2)	16 (20.0)	6.8	−10.9	−27.2	5.5	0.1845

CI, confidence interval; NIE, net intervention effect (difference in intervention area from baseline to endline minus difference in comparison area from baseline to endline).

### Clinical practice

The clinical management of rape improved significantly in the intervention area at final assessment. Significant improvement was noted in the pregnancy-related services [net effect 49.3%; 95% CI: (31.0–67.6), *p*<0.0001], STI-related services [net effect 51.2%; 95% CI: (33–69.3), *p*<0.0001], as well as the use of rape kits for the collection of forensic evidence [net effect 64.1%; 95% CI: (46.7–85.5), *p*<0.0001], as presented in Table 4.

**Table 4:** Health care workers’ clinical management of rape in the intervention and comparison areas at baseline and final assessment

	Intervention			Comparison						
						
	Pre *n*=98 (%)	Post *n*=89 (%)	Estimate of change (%)	Pre *n*=53 (%)	Post *n*=80 (%)	Estimate of change (%)	NIE	CI 95% Lower	CI 95% Upper	*p*
How can you identify survivors of sexual violence?										
Look at the wound	55 (56.1)	78 (87.6)	31.5	37 (69.8)	54 (67.5)	−2.3	33.8	13.2	54.4	0.0010
Ask the patients directly	81 (82.7)	80 (89.9)	7.2	43 (81.1)	57 (71.2)	−9.9	17.1	−0.8	35.1	0.0564
The patients tell	91 (92.9)	84 (94.4)	1.5	47 (88.7)	57 (71.2)	−17.5	19	3.7	34.2	0.0129
The patients’ relatives tell	83 (84.7)	76 (85.4)	0.7	41 (77.4)	58 (72.5)	−4.9	5.6	−13	24.2	0.5500
Other health care providers tell	67 (68.4)	67 (75.3)	6.9	26 (49.1)	44 (55.0)	5.9	1	−21.2	23.1	0.9302
How do you deal with the patients who are survivors of sexual violence?										
Keep them at the unit and deal with them	78 (79.6)	79 (88.8)	9.2	28 (52.8)	53 (66.2)	13.4	−4.2	−24.7	16.2	0.6775
Transfer/move them to other units/level	63 (64.3)	75 (84.3)	20	22 (41.5)	51 (63.8)	22.3	−2.3	−23.7	19.2	0.8331
Give PEP to survivors	85 (86.7)	82 (92.1)	5.4	39 (73.6)	50 (62.5)	−11.1	16.5	−2.2	35.2	0.0774
Seek survivors’ consent to HIV test in order to give PEP	80 (81.6)	71 (79.8)	−1.9	45 (84.9)	53 (66.2)	−18.7	16.8	−1.8	35.4	0.0713
What pregnancy-related services do you routinely offer the patient after rape?										
Pregnancy test	78 (79.6)	82 (92.1)	12.5	44 (83.0)	37 (46.2)	−36.8	49.3	31	67.6	**<0.0001**
Emergency contraceptive pills	66 (67.3)	87 (97.8)	30.4	31 (58.5)	43 (53.8)	−4.7	35.1	14.8	55.5	0.0005
What STI-related services do you offer the survivor after rape?										
Give prophylactic treatment	62 (63.3)	66 (74.2)	10.9	32 (60.4)	51 (63.8)	3.4	7.5	−14.5	29.5	0.4942
Refer to an STD/STI clinic	78 (79.6)	87 (97.8)	18.2	42 (79.2)	37 (46.2)	−33	51.2	33	69.3	**<0.0001**
Send swab to lab to test for STIs	58 (59.2)	81 (91.0)	31.8	30 (56.6)	27 (33.8)	−22.9	54.7	33.7	75.6	**<0.0001**
Do you collect physical evidence from survivors/victims (e.g. clothing, footwear, hair, fibers, or debris, etc.)?	46 (46.9)	69 (77.5)	30.6	14 (26.4)	21 (26.2)	−0.2	30.8	10	51.5	0.0030
Do you use a pre-packaged rape kit when conducting the exam?	22 (22.4)	73 (82.0)	59.6	9 (17.0)	10 (12.5)	−4.5	64.1	46.7	81.5	**<0.0001**
Does the hospital/health center have any specific regulations or guidelines on providing treatment services to the survivor of violence?	58 (59.2)	83 (93.3)	34.1	18 (34.0)	26 (32.5)	−1.5	35.6	29	64.3	**<0.0001**
Do you use the aforementioned materials?	37 (37.8)	78 (87.6)	49.9	8 (15.1)	18 (22.5)	7.4	42.5	24.2	60.7	**<0.0001**

CI, confidence interva; PEP, post-exposure prophylaxis; STD, sexually transmitted disease; STI, sexually transmitted infection; NIE, net intervention effect (difference in intervention area from baseline to endline minus difference in comparison area from baseline to endline).

### Recommendations given by HCWs

The majority of HCWs in the intervention and comparison areas felt resources were insufficient to support rape survivors. Most (91.0%) identified insufficient budget as the biggest problem at final assessment, compared with only 48.0% at baseline in the intervention area. More than 80% of HCWs in both areas admitted that local authorities have inadequate knowledge about GBV or that health care providers lack skills. Almost all HCWs in the intervention and comparison areas at final assessment showed an interest to participate in training programs on violence against women [89 (100.0%) vs. 78 (97.5%)].

## Discussion

To our knowledge, this is the first evaluation study in Tanzania to improve the health care response to sexual violence. The findings from this study revealed significant improvement in HCWs’ knowledge and clinical practice toward sexual violence after the training program. Although differences in length, content and settings, as well as evaluation designs have hindered clear comparisons between training programs, research has consistently found that training on violence against women improves awareness and increases the detection of survivors (20, 21, 28, 29).

Worldwide, clinicians within primary care and other health care settings are not responding adequately to sexual violence (14, 17). In this study, it is clear that capacity building of HCWs and the availability of support services are necessary ingredients for the survivors of sexual violence to address their short- and long-term health needs. The majority of HCWs admitted that resources were insufficient to support rape survivors. Therefore providing HCWs with guidelines, medications, and a forensic kit may have improved their knowledge as well as clinical practice with regard to rape management. This training program appeared to increase knowledge on sexual violence management in the moderate period of 10 months. Other training interventions report significant improvements in knowledge and attitudes about interpersonal violence in immediate post-training measures (30, 31) as well as when tested at 6- to 10-month follow-ups (32). On the contrary, evidence about long-term effects remains inconclusive (30).

Quantitative assessments of attitudinal changes after training are inconclusive due to lack of validated scales on violence (33) and contradictory findings in the field. Changes in attitude require a longer intervention and follow-up period; however, in this study, there was a trend toward reduction of accepting attitudes, although insignificant. Some interventions found significant lasting increments in favorable attitudes still remaining at 6 months or beyond (34), whereas other studies have found no significant changes immediately after training or at 6-month follow-up (33). As shown in the previous study (20) as well as in this study, most HCWs would welcome training. Willingness of HCWs to participate in training could be regarded as a non-accepting attitude toward violence.

Training interventions can also effect changes in health professionals’ practice. In this study, we found significant improvement in HCWs’ management of rape consequences. A majority of the HCWs had encountered a rape survivor and reported being able to identify and refer to a higher center whenever necessary. Existing literature has focused more on whether women should be ‘screened’ for violence, and the impact of an intervention on the number of cases detected (35–37). The majority of studies found that health providers who received training were more likely to enquire about domestic abuse compared with pre-training conditions (16–20, 38, 39) or to control groups with no training (32, 33). The number of rape cases that were reported to the community leaders and later referred to the health facilities in the same study area increased by more than 50% after community intervention as noted in the rape registries. However, for these improvements to occur, a supportive environment is critically important, and it must include factors such as access to guidelines or protocols as well as adequate material resources (13, 14).

Updated training workshops for health professionals are probably necessary to achieve the sustainability of the improvements generated by the training intervention. This could provide a secure base for addressing new challenges in the clinical field. However, sustainability of training in the long run is a common challenge. For instance, in this study, poor record-keeping and high staff turnover were some of the constraints faced within the program. Nine of the participants were transferred to other health facilities and therefore could not be reached for final assessment. To address the challenge of high staff turnover, the relevant ministries should develop a pre-service curriculum that would facilitate the training of all those who graduate through different programs. Further research is necessary to understand the successes and the operational lessons to be learned before upscaling in other settings. Qualitative research is needed to explore what the survivors want from the interventions and what outcomes they find beneficial, and to obtain measures of their physical and psychological well-being.

Strengths of this cross-sectional survey include the use of a comparison group to ascertain the knowledge, views, and practices of HCWs in relation to violence against women and children, as well as the high response rate of primary care clinicians. The study was conducted in rural settings; although we do not know how representative the findings are in an urban setting, research shows that rural service providers have poorer access to relevant training and report a lack of adequate resources within their locality (40). It is likely that the effect of the intervention would be lower in urban settings compared with rural settings because in urban settings health providers have more access to information and better resources. Moreover, staff training and on-site integration of services for survivors may have resulted in better coverage than interventions implemented at the tertiary level, as primary- and secondary- level facilities are closer to the community. One possible limitation of this study was the higher number of doctors in the intervention than in the comparison area. This difference in HCWs could be attributed to the geographical location of the Ulanga district, which is more remote, where doctors would be less likely to want to go and work. If anything, this may have resulted in an overestimate of knowledge and good practice among HCWs in those localities. Therefore, the findings cannot be generalized to the entire country. The HCWs’ clinical practice was examined by the use of self-reported rape registry books, as direct observations were not feasible due to the large geographical distribution of HCWs and poor road conditions. Provision of materials such as rape kits might have enhanced the good practice of HCWs. Nevertheless, it is also necessary to have clients’ perspective on the clinical practice of HCWs to be able to confirm the improvements gained by the HCWs. We are also aware that we conducted multiple testing which might have inflated the α error. Being a quasi-experimental design, this may have resulted in cluster effects.

## Conclusions

The current intervention may provide initial hints for the effectiveness of the training approach. Training on the management of sexual violence is feasible, and the results indicate improvement in HCWs’ knowledge and practice but not attitudes. Lessons learned from this study for successful replication of such an intervention in similar settings require commitment from those at strategic level within the health service to ensure that adequate resources are made available, such as commodities for caring for sexual violence survivors, protected time for staff training, and involvement with multi-agency sexual violence forum. In this sense, both a ‘top-down’ and a ‘bottom-up’ approach to the implementation of new policies and guidelines may be needed in order to bring about a shift in organizational culture.

## Paper context

This paper describes the changes in knowledge, attitudes and clinical practice towards sexual violence among health professionals, at primary care level after a training program. The results indicate improvement in healthcare workers' knowledge and practice but not attitude. Training of health professionals is feasible and provide first hints for the effectiveness of the training approach.
